# Mr. Travers on Diseases of the Eye, and Their Treatment

**Published:** 1821-06-01

**Authors:** 


### VIII.

*A Synopsis of the Diseases of the Eye, and their Treatment: to which are prefixed, a short Anatomical Description, and a Sketch of the Physiology of that Organ.* By BENJAMIN TRAVERS, F.R.S. Surgeon to St. Thomas's Hospital. One vol. 8vo, pp. 425, six plates, 32 beautifully coloured engravings. London, 1820.

" Vel me monere hoc, vel percontari puta:—

" Rectum est, ego ut faciam; non ut deterream." *Ter.*

WE believe we shall be credited when we declare that our analytical labours are generally performed "*con amore,*" and without any of those feelings of languor, dejection, and disrelish which must often oppress the author whose hard fate it is "to force a churlish soil for scanty bread." Yet we are compelled to confess, however reluctantly, that the perusal of some works puts our patience and our temper completely to the test, whether by generating a great flow of bile, and corresponding irritability of mind;—or by enveloping us in a kind of Beotian atmosphere that stupifies us for a time, until we are enabled to emerge into the cheerful sunshine of intellect once more. On the other hand, when we meet with a work of sterling merit, and at the same time, well arranged and well written, we enjoy at least the "feast of reason," if not the "flow of soul;" and it need hardly excite surprize if, in such cases, we prolong the genial banquet to a late hour, from a consciousness that such treats must be comparatively few in our literary journey through life.

We feel ourselves under deep obligations to the ingenious and able author of the work before us, and the profession will undoubtedly appreciate the valuable mass of facts and observations which Mr. Travers has here presented them from extensive personal experience—being, indeed, "the result of a more ample opportunity of observing the diseases of the important organ of which it treats, than commonly falls to the lot of hospital surgeons." This opportunity is derived from a period of seven years official situation as surgeon to the London Infirmary for Diseases of the Eye, besides a wide range of private practice. Our author is of opinion, that "the advantages obtained by the subdivision of professional talent and labour, are infinitely overbalanced by those which arise from the general and undivided application of these instruments of knowledge;" and he thinks this doctrine is strikingly illustrated by the prejudice arising to ophthalmic science in this country, from the confinement (formerly) of that branch in the hands of a few. This is now at an end, and the *respectable* exclusive oculist will be a name unknown in the next generation. The institutions for treating ophthalmic diseases are now conducted by such distinguished and zealous medical officers, and are disseminating ophthalmic knowledge so widely through the profession at large, that all mystery or pretensions to exclusive superiority must fall, as a matter of course.

The invaluable work before us is divided into three parts: the 1st, dedicated to the ANATOMY and PHYSIOLOGY of the eye and its appendages—the 2d, to the PATHOLOGY of the membranes, humours, and appendages of that organ; and the 3d, to the TREATMENT.

The anatomical portion occupies forty-four pages, and presents an animated, yet exceeding chaste and terse, delineation of all that is interesting to be known, or necessary to be remembered, of the structure of the parts, without wasting his own time, or perplexing the reader by uselessly dividing, and tiresomely naming the "ravellings of minute anatomy." Into this part of the work we cannot be expected to enter. About an equal space is devoted to the *physiology* of the eye and its appurtenances, and to it we may apply the same observation as to the anatomical section.

The first chapter of the SECOND PART of Mr. Travers' book embraces the pathology of the membranes, and is divided into five sections, viz. on the Conjunctiva—Cornea—Sclerotica—Choroid and Iris—Retina. These sections we shall notice in their order.

I. *Conjunctiva.* After some anatomical observations on the vessels supplying the conjunctiva, as derived from two sources, the palpebral and ophthalmic muscular arteries, but very freely inosculating with each other, our intelligent author remarks that, though in a tranquil and healthy state of the eye, little, if any, red blood is admitted into the superficial vessels, yet, under a very temporary excitement, the red fluid enters them on the sclerotic conjunctiva. It is wisely ordered, however, that the condensed connecting texture upon the cornea prevents the admission of red blood into *its* vessels, even under a very high degree of inflammation. Mr. Travers avers that the susceptibility to increased vascularity of parts, under excitement, "is in proportion to the quantity of cellular texture entering into their composition, or connecting them with subjacent parts." For an elucidation of this he refers to a comparison of the faucial and tracheal membrane with the pleura—the periosteum with the lining membrane of the veins and arteries.

The admission of red blood, then, into the *sclerotic* conjunctiva marks pretty accurately the progress of inflammation; while the first effect of this disease upon the *cornea*, is haze or dimness, depending on a loaded state of its serous vessels. This dimness is immediately removed by rebalancing the circulation, or removing the irritation destroying that balance. Transient dimness, in fact, is merely "a condition of congestion;" but, if continued for a certain time, produces a deeper and more permanent opacity, viz. effusion into the connecting texture, and thickening of the conjunctiva upon the cornea. The peritonæal, arachnoid, and synovial membranes, probably exhibit the transient and permanent opacity, in the distinct stages of congestion and effusion. The epidermis, in the state of blush and incipient vesication, forms a striking contrast to the above.

"The conjunctiva is to the cornea, what the periosteum is to the bone. It nourishes the superficial lamellæ; wherever it is completely detached, the exposed surface of the cornea ulcerates, and its vessels repair the breach. To pursue the analogy, the interlamellar texture of the cornea may represent the medullary membrane; gangrene therefore does not ensue but from a permanent destruction of both textures, as by blows and explosions, which mechanically disorganize; by the action of lime, gunpowder, strong acids, and other chemically destructive agents; or by the strangulation of the vessels of both textures, as in the excessive chemosis, which destroys on the same principle as the paraphymosis, or the strangulated hernia." P. 91.

The characters of *local and healthy* inflammation of the eye are well known, and exhibit the simplest example of the disease; but this phlogosis is much modified by certain constitutional causes or dispositions—as for instance, struma. In *this* case, (where it has not proceeded to change of structure) the vascularity is inconsiderable. It is marked by great intolerance of light, and, in its simplest form, is almost peculiar to children—stationary—marked by a very slight redness of the sclerotic conjunctiva—and the highest degree of morbid sensibility to light, which, Mr. Travers observes, is purely a disorder of function, never impairing the faculty of vision, though far exceeding that which accompanies the acutest inflammation to which the eye is liable.

"I attribute it," says this acute pathologist, "to a morbid sympathy of the retina with the secreting surfaces of the primæ viæ and the skin, for neither of these organs perform their healthy functions during its existence. The tongue, the index of the former, shews by various signs gastric irritation or disordered digestion, and the cutaneous surface is remarkably dry and harsh. Accordingly it is cured by diaphoretics, as tartar emetic to nausea, James's powder, or calomel combined with opium in small doses; by the warm bath; and materially corrected, if not removed, by a preternatural secretion in the vicinity, as by an open blister on the nape of the neck. I have often seen an aggravated intolerance removed in twelve hours by the application of a blister." 93.

Other organs of sense, as of the hearing and smell, are sometimes rendered morbidly acute, analogous to the above-mentioned state of the eye, and that without any organic change.

The nebula and pustule of the corneal conjunctiva are the terminations of this inflammation, when texture is affected—to which may be added the small herpetic ulcers, reddish brown points, giving to the cornea a scabrous appearance. Aphthæ or pustules are disposed to form on the sclerotic conjunctiva, at or near the verge of the cornea, and not dissimilar, in some cases, to those affecting the mouth, fauces, and intestinal canal. On the cornea, pustules are more rare, except in children; and, like the aphtha affecting the glans penis or fine cutaneous textures, usually terminate in an ulcer.

A puriform discharge, in ophthalmia, is furnished by inflamed meibomian follicles and the conjunctiva bordering them, in the same manner as the lacunæ of the urethra, and the mucous glands of the nares, fauces, rectum, vagina, &c. pour forth a similar fluid, when mildly inflamed—while in vehement acute inflammation of these parts the purulent matter is furnished by the villous surfaces themselves.

"The sclerotic conjunctiva in acute suppurative ophthalmia presents the following states. 1st. Serous effusion (œdema) which is common to other inflammations, and especially those of a less vigorous kind. 2d. Effusion of lymph (chemosis) peculiar to this form of inflammation, by which it acquires a solid augmentation of bulk. 3d. Villosity, or a subsequent prolongation of the extreme vessels in the form of villi, which secrete pus. The strict adhesion of the conjunctiva to the cornea prevents these changes from taking place upon that membrane. Upon the tarsi the conjunctiva thus affected becomes preternaturally vascular, thickened, and scabrous, or forms fleshy eminences. That the vascular villi of the conjunctiva secrete pus, may be ascertained by the aid of a lens. The pus, when formed, collects in the interstices of the villous texture. We have no evidence, as before observed, that the conjunctiva is a secreting surface in the healthy state." 96.

There is a mild, or intermediate form of this suppurative ophthalmia, affecting principally the conjunctiva palpebralis, while that reflected over the globe is simply intumescent. This form seldom injures the cornea, Mr. Travers thinks, but frequently leaves a granulated state of the conjunctiva palpebralis, as in the most acute form. The highly contagious character of purulent ophthalmia is well known. Our author thinks it happens twenty times under three months of age, for once after that period. Fluor albus or gonorrhœa in the mother—the transferrence of matter by towels, sponges, or any other vehicle, from one person or part to another, are the usual means of propagating this dire affection, which runs through armies, schools, and families; certainly contagious, but sometimes apparently epidemic.

Chemosis and ecchymosis have been confounded together by some writers; but, as will be seen in the last quotation from our author, they are widely different. It is after the effusion of lymph, in chemosis, so characteristic of purulent ophthalmia, that the conjunctiva forms "fungous excrescences, pendulous flaps, or hard rolls protruding between the palpebræ and globe, and everting the former, (ectropion,) if not protruding, causing the turning of the lid over against the globe, (entropion,)" The tarsal portion also takes on the hard granulated surface, productive of incessant irritation of the sclerotic conjunctiva, ultimately rendering the cornea opake. Various other morbid membranous growths, referrible to irritation, are described by our author at page 99, as also the fungous conjunctiva, a firm fleshy growth projecting from between the eyelids and globe, of an orbicular figure.

The conjunctiva, like the mucous membrane of other parts, is sometimes the seat of carcinoma, which is not the case, Mr. Travers thinks, with any other structure of the eye, excepting the lacrymal gland. The PANNUS, or chronic thickening, with opacity, of the sclerotic conjunctiva, results, Mr. T. thinks, from relaxation of its subjacent connecting tissue, whereby the membrane becomes redundant in extent, and folded in duplicatures on all sides of the cornea—somewhat analogous to the elongated uvula.

"The membranous pterygium is a true nebula of the sclerotic conjunctiva; the fleshy is an adipose or sarcomatous growth beneath the sclerotic conjunctiva. It extends from either canthus or sinus palpebralis, most commonly from behind the caruncula lacrymalis; and by its increase forcibly detaches the conjunctiva from the cornea. In its progress it occasions a permanent and indelible opacity by the thickening of the conjunctiva, and the deposition of lymph in the interspace of these membranes, in the form of a little tongue-shaped process. The wedge-like figure of the fleshy pteryx, and its gradual extension upon the cornea, afford the best pathological demonstration of the continuity of the conjunctiva; and the spread fan-like figure of the membranous, its semi-transparency as well as its termination in simple nebula of the corneal conjunctiva, shews the difference in the nature of the two diseases. Both this and the disease last mentioned, like other morbid growths of the cellular texture or beneath it, are most prevalent in warm climates." 101.

The encanthis, a morbid enlargement of the lacrymal caruncle, is extremely irritating, and occasions epiphora by a forcible diversion of the lacrymal puncta from each other, and from the surface of the globe. Mr. Travers has never known it assume a malignant character. The conjunctivæ palpebrarum and scleroticæ occasionally adhere by membranous bands, called FRENA or FRENULÆ, forming a troublesome and often an irremediable deformity. It is not necessary that both surfaces should be wounded, in order that adhesion may take place. "The opposite uninflamed surface, as Mr. Hunter observes, accepts of the union."

"I have seen these frena produced by a slit eyelid from a fall, and trifling as the inconvenience might seem, it so restricted the motions of the globe, and the disease was so materially aggravated by operations to relieve it, *i. e.* by the multiplication of frenula, that the patient became disturbed in his intellects from an exaggerated sense of his misfortune." 104.

The sclerotic conjunctiva being a *mucous*, and the corneal a *serous*, membrane, the *former* has a tendency to suppurative, and the *latter* to adhesive, inflammation. This is their pathological distinction.

II. *Cornea.* Ulcer of this texture begins, not in abscess, but in a circumscribed deposit of lymph, or in pure ulcerative absorption, without pus, and unaccompanied by any appearance of coloured vessels. The organizing process, however, is sometimes performed by these last. The appearance of coloured vessels upon the corneal conjunctiva may be referred to one or other of the following states, viz. 1st. to the presence of adhesive inflammation excited by a pustular ulcer of the cornea—2d, to the duration of acute strumous ophthalmia, rendering the serous vessels pervious to red blood—3d, to a state of chronic inflammation, in which, straggling solitary vessels, of a varicose appearance, run to one or more specks; or, proceeding from opposite sides of the sclerotic conjunctiva, traverse the opaque cornea, and freely anastomose upon it—a common sequel of purulent ophthalmia, whether accompanied or not with the granular conjunctiva tarsi. Many acute physiological and pathological conjectures are here introduced by our author, for which we refer to the volume itself.

The acute interstitial ulcer sometimes opens externally, by absorption of the conjunctiva and superjacent portion of lamellæ. Its figure is frequently crescentic, and traverses a part, or the whole diameter of the cornea. Upon close examination, the conjunctiva will be found to be absorbed at the part opposite to the ulcer, and the exposed scabrous surface of the cornea renders the motions of the upper lid acutely painful.

"The deposition of the adhesive track precedes the appearance of red vessels, which are derived to it in one or more fasciculi from the sclerotic conjunctiva, and by which its healing is perfected, as in the ulcer opening from the surface, before described." 115.

The acute interstitial ulcer, in debilitated habits of body, or when produced by considerable violence, instead of opening upon either surface of the cornea, spreads between its lamellæ—and if it occupy a large and central portion of the cornea, it usually terminates by slough of the entire membrane.

But when, as more frequently happens, the interstitial ulcer opens into the anterior chamber, HYPOPION is produced, a mixed secretion of lymph and pus—the former flaky and inorganizable, and situated exterior to the fluid.

When the cornea is perforated by external ulcer, the iris falls into the breach, and there becomes adherent, termed procidentia iridis.

"In the progress of an external ulcer to the interior of the cornea, and before it penetrates into the chamber, a remarkable appearance is occasionally presented, viz. a transparent vesicle, which fills the aperture, and is supposed to be the membrane of the aqueous humor. I have never seen this state maintained: the prolapsus iridis follows in a few hours, notwithstanding the use of the lunar caustic and other stimulants. This has led me to question its being a distinct texture, and its appearance corresponds accurately to that of the innermost lamella of the cornea, which after losing its support yields to the pressure of the humor, and assumes the vesicular form." 117.

Opacities of the cornea are of three kinds—1mo. thickening of the conjunctiva and effusion of adhesive matter between it and the cornea, or between the lamellæ of the latter—commonly the product of acute strumous ophthalmia. While recent it admits of removal by excitement of the absorbents, "especially by that which mercury produces." 2ndo a slow change of texture without breach, "similar to that by which the pleura, or choroid, or capsule of the lens is converted into bone." The yellow pearly opacity, resembling the inside of an oyster-shell, is of this kind. It generally results from continued, or frequently relapsing strumous inflammation, the layers of the cornea becoming opaque, indurated, and condensed, not admitting separation by the knife or maceration. 3tio. new matter supplying a loss of substance in the cornea, from ulceration or gangrene. Its figure is more abruptly circumscribed than the second variety, and bears a greater resemblance to a cicatrix. The more these specks vary from a white colour, the less chance is there of reducing them.

"The peculiar hue and loss of tension, as well as lustre, of the *dead* cornea in acute suppurative ophthalmia, has been aptly pictured by Mr. Saunders, by the terms 'cindery, ragged, flocculent.' It is important, because I have satisfied myself that the first change of the cornea in this disease is purely nebulous, produced by the deposition of adhesive matter; and if the inflammation be arrested even on the verge of gangrene, the cornea is susceptible of restoration by absorption. This fact I had lately an opportunity of establishing, in the case of a lady who was rendered blind by acute suppurative inflammation of the conjunctiva: so inevitable to all appearance was the destruction of the cornea, which had sloughed in a deep sulcus at its junction with the sclerotic above, that the most experienced practitioner of my acquaintance in this branch of surgery pronounced the case hopeless and irremediable, and took his leave. The highest tonic regimen, bark, wine, and opium, followed close upon a very active and bold depletion, and the anterior chamber was fortunately and unexpectedly preserved. No sooner was a sign of the arrest of sloughing ulceration obtained, than I commenced a mercurial course; in three days the system was affected; the recovery of the figure and transparency of the cornea was rapid and complete beyond all expectation, and an equally perfect state of vision was restored and established." 120.

Gangrenous opacities of the cornea from foreign substances, destroying its texture, are sometimes superficial, and a kind of exfoliating process takes place. More frequently the disorganization is complete. Recent opacities of a diffused semi-transparent character admit of absorption. Not so where the interstitial deposition has been abundant and of long standing—and the lamellæ compacted.

Mr. Travers has seen several cases where the conjunctiva was converted into a rugous and opaque skin, knitting the lids close to the globe. There is here no secretion of tears, and our author attributes the disease to an obliteration of the lacrymal ducts from chronic inflammation of the conjunctiva.

"All stimulant substances, not escharotic, applied to remove opacities of the cornea, act in the same manner as rubefacients upon the skin; they excite a temporary vascular action, which is followed by a correspondent excitement of the absorbents. I have often seen an opaque portion of the cornea cleared by a puncture with the couching needle. If the point of salutary excitement is exceeded, the increased vascularity is permanent, and occasions increased deposition. Injections applied to ulcers do not excite the absorbent action in the same ratio, but occasion a permanent increase of the vascular action, which is here below the ordinary standard. This instance of the adaptation of the same means to different ends, according to the state of the part, is perhaps the best practical illustration of Mr. Hunter's quaint but expressive phrase, "stimulus of necessity." 121.

Mr. Travers' pathological observations on staphyloma are very acute and interesting. We can glance at but a few of them. This distressing and unsightly complaint is of two kinds, viz. from dilatation and breach of the cornea. In the first, or spheroidal staphyloma, the effect of pressure is to thicken the remaining corneal lamellæ by a deposition of adhesive matter, as in the aneurismal and herniary sacs. In the second, or conoidal staphyloma, the recently deposited matter yields to the pressure *a tergo* before its organization is complete. Sometimes the two forms are combined. The remediableness of the deformity, by an operation, depends upon a sufficient portion of the iris being left—this last being kindly disposed to granulate. "Three or four days after the operation for staphyloma, the iris is seen coalescing with the conjunctiva, and throwing up fleshy pullulations, which, contracting into a little button-like eminence, seal up and permanently secure the crystalline and vitreous humors; thus the spherical figure of the globe is preserved to support the lids." If the section be posterior to the plane of the iris, the vitreous humor escapes, the globe collapses, and sinks in the socket.

There is a curious conoidal projection of the cornea, from thinning and absorption of its interlamellar texture, rendering vision inconveniently short, so that objects, at a very moderate distance, are much confused in their appearance. It is not preceded by inflammation or any assignable cause, and no remedy has yet been found for the complaint. "But a pupillar aperture set in a black ring frame, about a quarter of an inch or more in depth, greatly assists the patient by lessening the confusedness of his vision." The presence of adhesive inflammation seems to be the chief distinction between staphyloma and conical cornea.

III. *Sclerotica.* After some acute anatomico-pathological observations, Mr. Travers goes on to remark, that when inflammation of the conjunctiva is allowed to progress, the ciliary vessels partake in the action, and give signal of this participation by the well-known and remarkable appearance of a vascular zone at the margin of the cornea. The texture of the sclerotica, however, serves as a shield to the finer tunics, to ward off inflammation from without, as well as external violence. When inflammation has at length reached these interior tunics, the vision is affected in a much greater degree than appearances would lead one to suspect. When the sclerotica is inflamed, the vessels which pursue a straight course to the margin of the cornea are strongly distinguished. They have a somewhat darker hue than the areolar vessels upon the loose portion of the conjunctiva. Mr. Travers has seen occasionally a primary sclerotitis, unaccompanied by any affection of the iris, and with a very slight vascularity of the loose conjunctiva. The inflammation is not acute—sometimes accompanies, and is sometimes metastatic of rheumatic inflammation. It is often seen with eruptions and sore throat of a pseudo-syphilitic nature. This coat sometimes yields in spheroidal staphyloma.

IV. *Choroid and Iris.* When the vascular zone at the margin of the cornea, before alluded to as indicative of sclerotic inflammation, is accompanied with dulness of the humours, a spastic contraction, or a very sluggish and limited motion of the pupil, impatience of light, and dimness of vision, we may conclude that the choroid and iris partake in the inflammation. The above indications are still farther confirmed by the presence of an habitual aching pain in the globe of the eye, forehead, and orbit, together with hair-like red vessels on the iris, and specks of extravasated blood in its substance. Adhesive inflammation takes place between the fibres of this muscle; the pupil loses its thin flowing edge, and becomes stunted, and gibbous. Iritis of moderate acuteness is often unaccompanied by any other appearance of inflammation; there being no distinct deposit of lymph, its deposition being rather inferred from the fixedness or slight change of figure in the pupil, than actually demonstrated. In this form, there is such an evening, or early morning exacerbation of pain, as compels the patient to get up, and even totally to deprive him of rest.

"Sometimes the pain affects the whole corresponding side of the head. In other instances, it is confined to the eyeball and its immediate vicinity, as the forehead, and temple, and bones of the cheek. The sensation is sometimes that of pulsatile pain, marking every injection of the ophthalmic artery, as of the radial artery in a whitloe. A sense of continued pressure or constriction, as from extreme distension of the vessels, is the more common character of the patient's sufferings. In the vehement acute iritis, lymph is variously deposited upon the face of the membrane, in small tufts here and there, or larger tubercular masses. The pupil, in this case is usually much misshapen, being rendered angular at those points of the circle at which the deposit has taken place, or is most abundant. Its aperture is sometimes partially covered, and sometimes completely blocked by a deposit of lymph. The pain, in this state, is not always augmented in proportion. It affects more the head than the organ. The vision is nearly, if not quite extinguished. The appearance of a stratum of lymph, coating the face of the iris, with a turbid state of the aqueous humor, belongs to chronic inflammation, which tends to opacity of the capsule of the lens, and constriction of the pupil." P. 133.

*Primary* iritis, as from syphilis or mercury, is distinguished from the *secondary*, or that by extension from the conjunctiva, by sparing vascularity of the latter, and consequently more distinctly conspicuous vascular zone. The attack is more sudden, the pain in the region of the orbit and head commences with the inflammation, and is more severe—the vision is more quickly and completely bedimmed —the effusion of lymph is *en masse*, and the disfiguration of the pupil greater. The terminations of iritis, if unsubdued, are constricted or closed pupil, with opaque capsule —coadhesion of the iris and cornea, partial or entire—organic amaurosis, followed by disfiguration of the globe, and often by protrusions of the choroid and sclerotica.

Primary iritis, our excellent author remarks, is rarely seen unaccompanied or unpreceded by syphilitic, pseudo-syphilitic, or what are called mercurial symptoms ; yet Mr. Travers has seen it exist independently of these.

" But I have since had many additional opportunities of confirming the facts before advanced, that where mercury has been used in various ways before the iris was affected, and before the other symptoms appeared which were referred to its use ;—where the primary affection was either altogether questionable, or at most a gonorrhœa, or a superficial sore, which healed by a simple topical application— the iritis has yielded to the steadily supported influence of mercury upon the system, in a manner the most satisfactory ; and that no other remedy with which I am acquainted, was competent to this effect." 135.

Mr. Travers adds that he is unacquainted with any fact in medical surgery which ranks with this, in point of importance, whether we consider the urgency and frequency of the occasion, or the indispensible necessity, and almost unerring efficacy, of the remedy.

The iris undergoes changes of colour, texture, and figure, by a continuance of inflammation. For these we refer to page 136 of the work.

V. *Retina.* This is rarely the seat of inflammation; and Mr. Travers thinks that it is an error to consider intolerance of light as a sign of this affection. In the strumous ophthalmia intolerantia lucis is in excess, yet the retina is uninjured. The effect also (Mr. Travers avers) of inflammation upon a nerve of sense, is to produce direct palsy, not increased excitability. The first or predominant symptom of an inflamed retina is a sudden attack of "vehement dashing pain of the most distracting kind," extending from the bottom of the eyeball to the occiput, or in the reverse direction, and the supervention, within a few hours, of total blindness, with occasional sparks and flashes of vivid light. The pupil, upon inspection, is gaping and motionless, as in confirmed amaurosis, and the humours are thick and muddy. The external signs of inflammation are in no proportion to these symptoms.

In some cases *choroid* inflammation attends, and in these the pupil is not thrown open, but is motionless. Besides diffused vascularity of the conjunctiva, the straight ciliary vessels are remarkably turgid, so as to give a livid red hue to the sclerotica around the cornea. The pupil soon becomes plugged with lymph, or the whole iris bulges forward, changes colour, and the crystalline turns opaque. The pain is attended with an alarming kind of confusion, as though the patient were about to lose his intellects. When the internal signs of inflammation are less obvious, and the humours and internal tunics undergo a slight but complete disorganization in the progress of the disease, meteoric flashes are frequent, even after the inflammation has run its course, affording delusive hopes of returning sight to the unfortunate patient. Mr. T. has seldom seen an example of this inflammation affording time for the beneficial operation of remedies.

Of amaurotic affections Mr. Traves makes two classes, organic and functional—the *first*, comprehending all alterations, however induced, in the texture or position of the retina, optic nerve, or thalamus—the *second*, including suspension or loss of function in the retina and optic apparatus, whether depending on mordid action in the vascular or sentient system of the organ. As causes of the first class we may enumerate.

"1. Læsion, extravasation of blood, inflammatory deposition upon either of its surfaces, and loss of transparency of the retina.

"2. Morbid growths within the eyeball, dropsy, atrophy, and all such disorganizations as directly oppress or derange the texture of the retina.

"3. The state of apoplexy, hydrocephalus, tumors or abscesses in the brain, in or upon the optic nerve, or its sheath, and thickening extenuation, absorption, or ossification of the latter.

"As causes of the second,

"1. Temporary determination; vascular congestion, or vacuity, as from visceral and cerebral irritation; suppressed, or deranged, or excessive secretions, as of the liver, kidneys, uterus, mammæ, and testes; various forms of injury and disease; and sudden translations of remote morbid actions.

"2. Paralysis idiopathica, suspension or exhaustion of sensorial power from various constitutional and local causes; from undue excitement or exertion of the visual faculty; and from the deleterious action of poisons on the nervous system, as lead, mercury, &c." 141.

From the above we may easily see that organic, and many forms of even functional amaurosis are incurable; whilst it must be recollected that the functional cases, by continuance, lapse into organic disease. "Even under the continued suspension of function, much more the duration of a state of excitement, the power of the retina, as of other parts, gradually fades, and is at length exhausted."

Here our author makes many ingenious and highly interesting observations on both classes of amaurosis, as they are caused or influenced by different habits, professions, &c. and also on the different degrees of fortitude with which the dreadful loss of light is borne. For these particulars we refer to page 144 *et seq.* of the work itself.

When the seat of *organic* amaurosis is in the eyeball itself, there are generally some or all of the following phenomena, viz. preternaturally, or at least, fully dilated pupil, feeble in its contractions on the sudden application of light, or absolutely motionless; an appearance common to both classes, though not invariable in either—congestion of the superficial vessels, especially of the long fasciculi of conjunctival veins—peculiar bluish grey tint of the sclerotic coat—sometimes a bulging out of one or more sides of the globe, or loss of sphericity, the sides appearing flattened—a diffused turbidity or milkiness apparently of the vitreous humour, resembling the humours in the eye of the horse, and termed glaucoma by the ancients, often mistaken for incipient cataract. "It appears deep-seated, diffused, and of uniform density; and in examining some such cases at long intervals, I have not found the appearance vary." The lens remains transparent. Mr. Travers acknowledges, however, that there are cases of deep-seated opacity so closely resembling that of incipient cataract, that it becomes next to impossible to decide on the actual state of the lens. A more common phenomenon in amaurosis is a white or greenish yellow spot, apparently in the fundus of the eye, and a little to one side of the visual axis, sometimes having a splendid disc resembling the tapetum of the sheep, or the coloured choroid of fish; but more usually occupying a circumscribed annular space, and is seen only in a strong light, and in a particular direction. The causes of this point are involved in obscurity. However, it is by no means a constant appearance in amaurosis, nor is it incompatible with useful vision.

"In the amaurosis from inflammation of the choroid or retina, where the diseased action has entirely subsided, the veins of the conjunctiva are varicose, the iris is discoloured, thick, tough, inelastic, and preternaturally vascular; the substance of the crystalline is more or less absorbed, or converted into a fluid, and discoloured; the vitreous humor is opaque and of a deep yellow colour. The retina, like the other transparent textures, becomes opaque under inflammation, and it is probable that under these circumstances, adhesive matter is effused upon the interior of the choroid; this supposition I have never had an opportunity of verifying by dissection, in cases of which the history was known." 150.

Our author here relates two curious cases; one of absorption of the vitreous humour and collapse of the retina; the other of amourosis, from cerebral tumour. Many cases of amaurosis from concussion have fallen under the inspection of Mr. Travers; and also of congenital organic amaurosis, for which we must refer to page 152 *et seq.*

At page 155 Mr. Travers takes up the subject of functional amaurosis, which he divides into three species, viz. 1^mo.^ the *symptomatic*, or that which is merely a symptom of some general disease, or disordered state of the system, as of general plethora, general debility, &c. 2^ndo.^ The *metastatic*, produced by sudden transference of morbid action from another organ of the body, as from the skin, testicle, &c. 3^tio.^ The *proper*, depending on a peculiar condition of the retina, as visus nebulosus, muscæ volitantes, &c.

*Symptomatic Species.* This, like nervous deafness, sometimes follows typhus, scarlet fever, infantile fever, and other acute constitutional diseases. It is sometimes a consequence of chronic wasting diseases, in which organic changes interrupt the nutrition of the system. Mr. Travers has seen a rapid and severe salivation, instituted for a remote affection, produce gutta serena of both eyes. The state of the circulation has a marked influence upon imperfect amaurosis, as well as upon nervous deafness. Mr. T. knows patients whose vision is benefited in a high degree, and others in whom it is as much deteriorated by the quickened circulation of a full meal and a few glasses of wine. The former, as may naturally be supposed, are spare and meagre; the latter plethoric. Of the mental emotions, grief appears to be most influential in producing symptomatic amaurosis. This affection is often seen in young widows. The amaurosis lactantium, in which the infant preys upon its mother, exhibits a familiar example of the disease from constitutional debility.

"Amaurosis depending on vascular congestion is marked by some or all of the following symptoms, viz. dilated and sluggish or immoveable pupil, ptosis, or strabismus, and oblique or double vision of the affected eye; a preternatural action of the carotids, flushed face, sense of weight, pain, or stricture of the scalp, lethargy, occasional tinnitus aurium, with greatly disordered and irritable stomach. The patient frequently complains, particularly in straining, stooping, or on first lying down, of seeing luminous sparks or flashes, and a reflection of one or more of the choroidal vessels, the visible pulsation of which is a cause of much distress to him. A person thus affected accurately described to me the zona minor iridis, as distinctly presented to his view." 158.

Undue determination of blood to the head often exists independent of general plethora, "and is aggravated by loss of blood," of which Mr. Travers relates an example in the person of a young medical gentleman, who came to him from the country in extreme anxiety, and solicited him to apply a ligature to the carotid artery. This gentleman was of short stature, and constitutionally healthy. His pupils were large, his countenance suffused, and bearing the appearance of preternatural determination of blood to the head. He had had two inflammatory attacks in the April and October of the same year; during which he had lost upwards of an hundred ounces of blood. He had now a constant heavy pain in the head, chiefly over the coronal suture, and in the direction of the sinuses, with tinnitus of the left ear.

"After stooping the giddiness was extreme, and a golden coloured spot, edged with black, appeared floating before the eye. He had been troubled with muscæ in excess, for a year and a half past; he had now fire sparks flashing before the sight, and saw a pulse in the choroid synchronous with that of the wrist. When looking at near objects he was not troubled with muscæ, but they were always numerous, in proportion as the object was remote. He did not complain of much dimness. His complaints were not relieved by topical blood-letting. He recovered gradually, but perfectly, under a regulated diet, and a course of the blue pill with saline aperients." P. 160.

The amaurosis from depletion is sometimes mistaken for that from plethoric congestion, owing to the coincidence of a dilated and immoveable pupil, muscæ, and a deep-seated pain in the head, with occasional vertigo, together with its occurrence often in corpulent habits. It supervenes somewhat abruptly on uterine floodings, and large and sudden depletion for acute diseases. The pain is not confined to the region of the orbit, but is that peculiar nervous pain to which women are subject after uterine hæmorrhage, attended with a sense of defined pressure, "as of an iron finger on the brain," and sometimes a distressing jarring noise, like that of a mill or threshing floor. "By a cautious use of tonics it is relieved—by whatever lowers or stimulates, whether diet or medicine, it is decidedly aggravated." The vision, in this form of amaurosis, our author observes, is further enfeebled by the loss of as much blood as flows from two or three leech-bites. This, he avers, is not imaginary. He has seen distinctly marked cases of it, "in which large and copious venesection was still urged as the only resource of art." This he considers to be a fatal mistake. We have seen several cases where local congestions of blood in the head, lungs, and other organs, appeared to depend on some peculiar state of the system which caused the blood to concentrate in these parts, while some other parts were deficient of the proper quantity. Here neither general nor local depletion did good. But we have seen decided advantage from revulsive measures, quietude, and regulated regimen. For amaurosis sympathetic of irritable conjunctiva, we must refer to page 161 of the volume itself.

*Metastatic Species.* Mr. Travers has seen this species of amaurosis from the state threatening effusion into the chest—from gout in the foot—swelled testicle; in all which cases the oppressed organ was as suddenly relieved as the eye was affected. "Thus a person goes to bed with good vision and rises blind." Here our author relates cases in illustration.

The gout attacks the eye through the medium of the stomach; vomiting occurring, with gastric pain, on the subsidence of inflammation in the extremities, succeeded by violent head-ache, with sudden and permanent loss of sight. To this class also belong the cases of amaurosis consequent upon the sudden suspension of the catamenia, or habitual hæmorrhoidal discharges—the rapid healing of large ulcers —the sudden retrocession of cutaneous eruptions.

*Amaurosis proper.* The following case will best explain a temporary palsy of the retina from over excitement. It is given in the words of a young gentleman ardently engaged in the study of the profession when thus interrupted.

" 'Having habituated myself for the preceding twelve months to intense study, reading and writing to a very late hour, which had been only interrupted for a few days by a slight inflammation of my right eye, I quitted London to recruit my health in the pure air of ———. This daily improved, but I found a growing imperfection in the vision of my left eye, which advanced unaccompanied by inflammation, pain, or any other external symptom of disease. It seemed at first a film before the sight, but at length amounted to a total loss of vision. On examination, I found the pupil greatly dilated, and learned that the iris had little or no action. By the advice of Mr. T. whom I now consulted, I applied a blister, extending from the centre of the forehead round the eye to the root of the nose. This drew well, and I continued it open for ten days, closing the eye from light during that period. I took at the same time a calomel and opium pill thrice a day. In the space of a few days my mouth became sore; the pupil acted, though unequally, and I experienced a gradual recovery of vision. In the course of six weeks, I was enabled to resume my studies, and could perceive no defect of vision. I had gradually reduced the dose of calomel, and now discontinued it, drinking the decoction of sarsaparilla. At the distance of four months from this occurrence, the pupil is regular and active, and the sight unimpaired.' " 165.

*Symptoms of Amaurosis.* Pain in the head and temples is a precursory symptom of amaurosis, diminishing as the dimness increases, and usually ceasing altogether when the amaurosis is complete. If, however, the pain be severe, remitting imperfectly, and increased by exercise, whether diffused or circumscribed in extent, it is usually connected with an *organic* cerebral change. In *this* case, derangement and torpor of the primæ viæ, loss of strength and flesh, disposition to stupor, occasional confusion of intellect, inaptitude to exertion, and paralysis of one or more muscles, will be concomitant symptoms.

" There is an intermittent spasmodic pain accompanying some cases of amaurosis, shooting through the orbit into the head, of the most acute and distressing severity; it makes a periodic attack at or about the same hour, every night, or every second night, and continues for several hours; it is accompanied with convulsive quivering of the muscles of the eye and eyelids, and profuse lacrymation; there is nothing in the appearance of the organ to explain its nature and origin." 168.

Mr. Travers believes it to be a *tic douloureux* affecting one or more of the orbitar branches of the fifth pair of nerves. He has cured two cases by arsenic, where opium failed to prevent the paroxysm. Paralysis of different muscles, as the levator palpebrarum, orbicularis, &c.—vertigo—ptosis—loss of association and direction—and strabismus, are all more or less symptomatic of amaurosis, and on each of these our author makes acute and interesting observations. The same may be said of Mr. Travers' remarks on hemiopsia—distorted position of objects, and muscæ fixed or floating. When these last are fixed, they usually belong to organic amaurosis—when floating, to functional sympathetic or proper amaurosis. In the fixed musca, the opaque spot varies in density, in different individuals, "and under a long but gentle mercurial course, I have known it become considerably less dense, so as not to intercept bright light."

The musca volitans is sometimes solitary; but more frequently an immense assemblage, descending in a cloud as the eye is raised, and ascending as it is depressed. The patient's attention being minutely directed to these spectra, they are curiously and minutely described.

" Almost every person has, at some time or other, seen these appearances, but especially those subject to dyspepsia, and disordered function of the stomach and liver. At the moment of approaching deliquium, they appear in one vast cloud, and they are harbingers of the intense bilious head-ache. At the instant of their appearance, the sentient extremities upon the fingers and tongue are so benumbed, that objects of touch and taste convey a very indistinct impression, as if some muffle were interposed. These sensations I am describing *ad vivum*, for I was formerly often the subject of this attack, which was followed by a certain degree of confusion of intellect, and temporary suspension of memory, so as greatly to embarrass, if not to take away the power of intelligible expression. I mention these opposite and transitory states of emptiness and plethora concomitant with the floating muscæ, to shew the purely functional origin of the affection. The one (deliquium) is an uninjected, the other (sick head-ache) an over-injected or congested state of the nervous texture; or suspension from vacuity, and suspension from plethora. An analogy is plainly to be perceived between the corresponding states of the sentient and visual extremities, described in the last affection, to that of a temporary incomplete paralysis." 177.

Coloured spectra, or luminous impressions of objects remaining upon the retina, are usually preceded by the fixed muscæ, and may then be regarded as a more advanced stage of the complaint, though sometimes Mr. T. has known them to be symptoms of functional derangement, and to disappear as the vision recovered. For a very curious and interesting case in illustration, we refer to pages 179-80-81 of the work.

A very frequent and characteristic symptom of functional amaurosis, our author observes, is a thin mist, fog, smoke, or gauze, which takes off the *acies oculorum acer*. Thus the letters of a book run together, and the outline of all minute objects is indistinct. This affection is unaccompanied with irritable conjunctiva, or tendency to effusion. It is seldom relieved by glasses—never permanently. The iris appears irritable and unsteady, often contracting quickly, but vascillating between contraction and dilatation, without a change of the light.

Another functional affection is an oscillation or wavering of objects, occasioning a dazzling and confused perception—sometimes resulting from simple congestion, but occurring sometimes in Mr. Travers's experience, without any sign of this state, and in persons of an opposite frame and temperament. In functional amaurosis there is sometimes a loss of the adjusting power, and consequently of distinct vision at different distances. That gradual abridgment of the focal range, occurring in advanced life, and requiring convex glasses, Mr. Travers refers rather to a loss of power in the retina than to a permanent change in the figure of the globe, on which it is usually supposed to depend. The same taking place, more abruptly, in early or middle life, Mr. T. considers a symptom of amaurosis.

"Ordinary observation proves that the effect of wear and tear is to allow of good distant vision, in which the parallelism of the rays of light supersedes the necessity of adjustment, while the near sight which requires the active or tonic state of the adjusting faculty, is impaired or lost. But if, as sometimes happens, the vision of near objects remains good while the distant is obscured, the evidence of the faultiness of the retina is direct. The correction of a defective adjustment by the use of glasses, in either case, proves no more than that the retina is not organically affected, while the failure of this corrective, which is frequent in the cases referred to, demonstrates the functional debility of the retina. In most of these cases the use of glasses is of temporary benefit, but if continued, it is followed by uneasiness or pain in the eyeball." 184.

Many other phenomena of impaired vision are alluded to at pages 184 and 185 of the work, to which we refer. Many curious and important observations follow on the influence of light on amaurosis, according to the state of the retina, and pupil, &c. Some people affected with imperfect amaurosis see clearest on first waking in the morning, others the reverse. In such cases the state of the stomach has an obvious influence. Emptiness will produce muscæ and a temporary blindness. In many amaurotic patients vision is clearest in the evening, appearing to have gained strength by exercise. These differences, Mr. Travers observes, are mostly referrible to the various susceptibility of the retina determining the requisite degree of illumination of objects for vision, and the adaptation of the pupil to that purpose. The cases of day and night blindness present the opposite extremes of variation in susceptibility of the retina, and these must be regarded as cases of proper functional amaurosis. The remarkable efficacy of blisters upon the temples in these cases confirms this fact. Our intelligent author has had abundant opportunities of ascertaining the influence of trades in producing or aggravating amaurotic affections. Tailors and shoemakers commonly remark that they never see so well as upon a Monday morning, which they justly attribute to the repose of the organ during the preceding day.

The action of the iris, in its pupillary movements, is, generally speaking, the surest indication of health in the retina. The mean state of the pupil is that of dilatation, where an amaurotic affection exists. When the perception of light fails altogether, the pupil is generally fully dilated and quite motionless.—In other cases it is not perfectly circular.

"The activity of the iris requires the free and uncompressed state of the retina, iris, and ciliary nerves. In the various forms of amaurosis, its activity is proportioned to the degree of integrity which these several parts retain, and the intensity of the stimulus. If the retina be opaque, compressed, or unsupported, the iris mechanically disordered, or the ciliary nerves palsied, the pupil is inactive, independently of the state of vision. In the first of these cases, it is evident the vision will be lost; but we continually see useful vision combined with the second and third, as after operations in which the iris has been half destroyed, or has become permanently adherent, or in malformations where it is half wanting; and in paralysis of the ciliary nerves accompanying the state of ptosis. But how shall we explain the activity of the iris in a state of absolute blindness? a case by no means uncommon. We can only explain it by concluding the organ to be sound, and the cause of the amaurosis remote, or at least external to it. Its motions in such a case are purely involuntary; the mental perception being suspended or annihilated. All that is required to excite them is the impingement of the ordinary stimulus upon the unchanged retina, the white sheet upon which the images of objects are impressed, the instrument, not the organ of perception. The iris, in such a case, acts by a sympathy independent of the brain." 188.

Thus, in two young ladies the iris was even vivacious, though blindness was complete, as was the case where a circumscribed tumor compressed the left optic nerve, immediately behind the ganglion opticum, producing total blindness. In cases of perfect amaurosis, where the pupil retains its ordinary size, but is motionless, (a circumstance by no means rare) Mr. Travers thinks that the retina has most probably undergone a change of texture. The ciliary nerves are uncompressed, as may be inferred from the undilated state of the pupil, but the source of their excitement, sympathy with the retina, is destroyed.

In concluding the subject of amaurosis, our author introduced a curious fragment from Milton, namely, his letter to Thevenot, a celebrated French oculist, describing the symptoms and progress of amaurosis in his own case, from functional debility to the confirmed, perhaps organic gutta serena. As the letter is not long, we shall give it a place here, out of respect to the memory of our immortal bard.

"Decennium, opinor, plus minus est, ex quo debilitari atque hebescere visum sensi, eodemque tempore lienem, visceraque omnia gravari, flatibusque vexari; et mane quidem, si quid pro more legere cœpissem, oculi statim penitus dolere, lectionemque refugere, post mediocrem deinde corporis exercitationem recreari: quam aspexissem lucernam, Iris quædam visa est redimere: haud ita multo post sinistrâ in parte oculi sinistri (is enim oculus aliquot annis prius altera nubilavit) caligo oborta, quæ ad latus illud sita erant, omnia eripiebat. Anteriora quoque, si dexterum forte oculum clausissem, minora visa sunt. Deficiente per hoc fere triennium sensim atque paulatim altero quoque lumine, aliquot ante mensibus quam visus omnis aboleretur, quæ immotus ipse cernerem, visa sunt omnia nunc dextrorsum, nunc sinistrorsum natare; frontem totam atque tempora inveterati quidem vapores videntur insedisse; qui somnolentâ quâdam gravitate oculos, a cibo præsertim usque ad vesperam, plerumque urgent atque deprimunt; ut mihi haud raro veniat in mentem Salmydessii vatis Phinei in Argonauticis:

——— *κάρος δε μιν ἀμφεκάλυψεν*
*Πορφύρεος, γαῖαν δε πέριξ ἐδοκησε φερεσθαι*
*Νειόθεν, ἀβληχρῷ δ' επι κώματι κεκλιτ' ἄναυδος.**

Sed neque illud omiserim, dum adhuc visûs aliquantulum supererat, ut primum in lecto decubuissem, meque in alterutrum latus reclinassem, consuevisse copiosum lumen clausis oculis emicare; deinde, imminuto indies visu, colores perinde obscuriores cum impetu et fragore quodam intimo exilire; nunc autem, quasi extincto lucido, merus nigror, aut cineraceo distinctus, et qausi intextus solet se affundere: caligo tamen quæ perpetuo observatur, tam noctu, quam interdiu, albenti semper quam nigricanti propior videtur; et volvente se oculo aliquantulum lucis quasi per rimulam admittit."

"LEONARDO PHILARÆ, ATHENIENSI:

"Septemb. 28, 1654."

---

* " ——— Vertigo vero ipsum circumdedit
" Atra, et terram opinatus est circumagi
" Ab imo, in languidum vero soporem delapsus est elinguis.
BECK'S APOLLONIUS RHODIUS. Lib. 2. v. 203.

### PATHOLOGY OF THE HUMORS.

I. *Aqueous Humor.* Hydrophthalmia, or a simple redundancy of the aqueous humor, is a sequel of chronic inflammation affecting the interior texture of the eye. The figure of the globe is preserved, but the distended sclerotic has a dark blue tinge. The cornea is extended and prominent, the pupil dilated and inactive, and vision inconsiderable, if not extinct. In some cases there is loss of figure of the globe, and staphylomatous enlargement of the cornea, which is speckled or exulcerated, frequently presenting fasciculi of red vessels on its surface—the result of an acute disorganizing inflammation. The bulged and transparent cornea gives the appearance of redundant aqueous humor; but it is only an increased capacity of the chamber—a distinction of importance, our author thinks, since the treatment of this and hydrophthalmy proceeds on opposite principles.

The aqueous humor is rendered turbid by inflammation of the choroid and iris, resuming its transparency when the inflammation ceases. It is well known that, when discharged by accident or operation, it is reproduced in ten or twelve hours. The solvent power of the aqueous humor over the exposed fragments of the crystalline lens is not, Mr. Travers thinks, superior to that of water, which is very slow. The rapid removal therefore of the fluid and flocculent cataract, when dissipated in the chamber, cannot, Mr. T. thinks, be explained on the principle of the aqueous humor possessing a solvent power superior to common water.

"The fragments of the lens have no more power of resisting absorption than an extraneous substance, and the process improperly termed, solution, is essentially referrible to the operation of the absorbents. The secreting function of the chamber is evidently a powerful one, from the reproduction of the humor in the course of a single night. That the absorbent function is nearly equal to it, is proved by the facts above-mentioned, but still more strikingly, by the rapid diminution and removal of the matter effused under inflammation, when quickened by the excitement of mercury." 197.

II. *Vitreous Humor.* The absorption of this humor is evident in cases of floating cataract, and in some forms of organic amaurosis, &c. Mr. T. examined the decayed eyes of horses, and found in many of them the opaque lens sunk in the vitreous chamber, and the vitreous humor almost entirely absorbed, the eye being filled with a morbid accumulation of aqueous humor. In some cases the globe was greatly enlarged and flaccid, resembling an undistended dropsical cyst, the crystalline being opaque, of its natural size and firm, and sometimes its capsule thickened and scabrous, bearing marks of inflammation, and, instead of the healthy vitreous humor, containing a yellow gellatinous fluid.

The tremulous iris, Mr. Travers believes to be connected with a relative disproportion in volume of the vitreous humor, whether congenital or accidental.

> "Couching and the operation by absorption, if roughly performed, break down a portion of the vitreous cells, which become obliterated; hence the frequency of floating cataract and tremulous iris after these operations. The loss of a very considerable proportion of the vitreous humor may take place without permanently impairing the vision, except of minute objects, as is proved by the successful issue of some cases of extraction, in which this accident has happened." 199.

This humour assumes a deep yellow or chocolate brown colour, frequently, in organic amaurosis, with or without cataract, and especially accompanying diseases of the iris. From the rapid and uninterrupted egress, in this and other cases, there is reason to infer that the cellular contexture is broken down, since this uninterrupted egress does not take place in the healthy humor. Mr. Travers has known blood to be effused into the cells of the vitreous humor within twelve hours after the operation of extraction, in consequence of straining on the commode, which was instantly followed by a severe pain darting towards the occiput. The coagulum was visible to both patient and surgeon; the former describing it as a central circular spot, intercepting the light. It was absorbed in the course of time, the patient gradually recovering tolerable vision. In another case, not occasioned by any improper exertion, there was active and continued hæmorrhage, producing excessive distension of the globe, attended with exquisite pain. These symptoms took place on the evening of the day of operation, and on the following day the humor, loaded with an enormous coagulum of blood, protruded at the section. Our experienced author has met with other cases in which hæmorrhage into the vitreous cells occurred in consequence of a blow, followed by inflammation and swelling, with sloughing of the cornea and protrusion of the humor, in the form of a large spongy mass, attended with severe pain in the head for two or three inches round the orbit. The occasional hæmorrage is profuse, and it is not improbable that, at one period of this disease, its aspect and character have led to the opinion of its being of a malignant nature. The eyeball ultimately sinks, with a total loss of figure.

The vitreous humor is subject to a complete change of consistence, and a total loss of transparency, the texture of its cells, and its volume and figure remaining. It is converted from a transparent albumen into an opaque substance resembling curd. Although the opacity is visible, the appearance differs widely from that of cataract.

"While the crystalline remains transparent, the same bright-coloured appearance is seen at the bottom or sides of the eye, which is supposed to announce the incipient medullary fungus. In the progress of the disease also, as in the malignant disease, the lens appears to become opaque, and is protruded so as forcibly to dilate the pupil; this becomes fixed, its edge roughened by detached pigment, and the iris convex, so as to give a conoidal figure to the globe." 202.

Several years ago Mr. Travers extirpated an eye in a fine infant, in whom this disease was supposed to be malignant fungus in its nascent state. The child has grown up healthy, the other eye remaining sound. Upon section of the eye, the vitreous humor presented the appearance above described. The tunics were all entire. Mr. Travers has since seen several cases of a convex and permanently dilated pupil, with a deep-seated opacity of a splendid yellow tint in children; these appearances, to his surprize, continuing stationary for years, unaccompanied by any disorder of the health. He therefore regards these cases as simple and uniform conversion of the substances of the vitreous humor, by an altered action of the secreting vessels, wholly independent of a malignant character. Yet he confesses that, unhappily, we possess no accurate means of diagnosis between malignant fungus and this disease, in their incipient state; nor do we know that the disease may not, sooner or later, take on an active and malignant character, as now and then occurs in the testicle, female breast, and other textures.

"There are, however, two marks of distinction sufficiently strong, between the malignant fungus and this disease of the vitreous humor; viz. the progressive or stationary condition of the disease, denoted by the state of the tunics and the eyeball generally, and secondly, the presence or absence of pain and constitutional irritation. To these I might add, especially as regards children, the affection of one or both organs, as affording a strong presumption that the disease is harmless in the first case; in the second, a conclusion that it is malignant." 205.

It is remarkable that both the disease under consideration, and the malignant fungus of the eyeball, are of most frequent occurrence in infancy. Mr. Travers has not seen the former in the adult.

*Crystalline Humor.* Excepting in one case, Mr. Travers has not witnessed any other result of inflammation of the crystalline lens and its capsule, than opacity. In this exception suppuration took place within the crystalline capsule, after a severe blow on the globe of the eye. Under the action of mercury the pus and lens were absorbed together, the case terminating in capsular opacity, with a constricted and misshapen pupil, and coadhesion of the iris and cornea.

The capsule of the lens will readily unite, when incised, unless a portion of the lens itself intervene. The capsule, when adhering to the iris, receives delicate red vessels, running in peduncles of lymph, produced from the inner border of the pupil. Small flaky portions of the pigment are also frequently detached on the margin of the capsule—an appearance commonly seen in the constricted pupil after chronic iritis, and often the result of reiterated attacks of inflammation, at short intervals, to which a constricted state of the pupil predisposes.

"The iris is much thickened by repeated depositions of lymph, until its texture becomes quite altered. There is a very imperfect and deranged state of vision, according to the degree and extent of opacity of the capsule, which admits of no improvement by the direction of the light; and sometimes a marked and painful determination of blood to the head. Except in this case, and in punctured wounds, the capsule is seldom partially opaque, but though its opacity is diffused, it is often not of uniform density, so that it has a dotted or mottled appearance. When calcareous matter is deposited, it is in small flakes or scales, which have a brighter tint than the opaque membranous portion. The opacity of the capsule, as of the cornea, varies in degree, from the slightest nebula to the opacity from change or conversion of texture. The incipient nebulosity is often, as before observed, difficult of discernment. Where the capsule is completely opaque, the lens undergoes a slow absorption; the capsule, however, remains transparent in most cases of senile cataract, not preceded by inflammation. The capsule, like all other textures of the body, undergoes absorption, when detached." 208.

The fluid, floculent, caseous, and hard cataract, are the principal and distinguishable degrees of density to which the opaque crystalline is subject. On these varieties Mr. Travers has written fully in the fourth and fifth volumes of the Medico-Chirurgical Transactions.

The opacity of the posterior capsule; *i. e.* the tunica hyaloidea, is very rare, which it would not be, Mr. Travers thinks, if the lens were invested in a capsula propria, especially after the operation of extraction, in which the anterior capsule only is lacerated, and the lens allowed to escape. Where it is met with, the lens and anterior capsule are usually transparent; and when this is *not* the case, and the cataract escapes with a posterior fold of opaque capsule, it is always, in our author's experience, accompanied with a considerable discharge of vitreous humor.

*Cataract.* The formation of cataract, though uncertain, is usually slow. A cloudy, semi-opaque state, or distinctly nucleated opacity sometimes remains stationary for years, or even for life; yet it *occasionally* forms with rapidity, without inflammation. Generally, however, the rapid formation of cataract is attended with inflammation, or preceded by diseases of the textures. The residence of a perfect cataract in the eye, is injurious—at least it is attended with the risk of destructive inflammation. The vitreous humor undergoes a partial absorption, and the lens, losing a proportional support, bulges forward, and presses upon its capsule and the iris—causing what operators call a narrow anterior chamber.

"Where the capsule yields from a blow or by absorption, and the pupil is dilated by the protruding lens, a violent inflammation, attended with very acute pain, is the invariable consequence. I have known this happen suddenly and independent of external injury, where the formation of the cataract has been gradual, and unattended with pain; and the spontaneous occurrence, though not so frequent, is precisely similar to that produced by the too free laceration of the capsule with the needle." 211.

The absorption of opaque lens is quick in proportion to the looseness of its texture, and its complete exposure, by breaking up and detaching its fragments, to the operation of the aqueous humor, particularly if there be a plentiful healthy secretion of the latter; for a turbid state of it indicates the arrest of its secretion, and *vice versa*. In confirmation of this, Mr. Travers observes that, in all cases of narrowed anterior chamber, by the partial coadhesion of the iris and cornea, consequent on injury or inflammation, the absorption is slow. Absorption does not take place, he says, at all, during the existence of inflammation, in which state the aqueous humor is in a morbid condition; and if the inflammation be deep-seated and protracted, the vitreous humor partake of it. Mr. Travers has seen a case of dislocated lens occupying the anterior chamber, followed by inflammation of the iris, from which membrane it derived an adventitious capsule organized by coloured vessels.

IV. *Diseases affecting the Eyeball.* Our author properly passes over the injuries, from external violence, to which the eye, in common with all other parts of the body, is liable, as coming within the range of general surgery.

Suppuration of the eyeball follows sometimes (though less frequently than from injury) a long continued and exasperated inflammation of the interior tunics. The globe becomes rapidly enlarged, protruded, and tense;—the conjunctiva, highly tumid and vascular, is rolled out upon the cheek, everting the lower eyelid—the pain is very acute and lancinating through the eyeball and head—the patient's health suffers, and the symptomatic fever is considerable. Pus collects in quantity—the cornea turns opaque, and slowly ulcerates or sloughs, giving vent to more or less of the contents of the eye, when the irritation gradually subsides. The eyeball afterwards shrinks up, and the cornea is obliterated. The hypopion or purulent secretion filling the anterior chamber, and originating from internal ulcer of the cornea, is not accompanied by the enlargement, acute pain, or irritating fever, marking abscess of the globe. The result, however, is the same—the perishing of the cornea.

Mr. Travers formerly supposed, what is generally believed still, that the bulb or globe of the eye was liable to be affected with cancer, but he is now of a different opinion—he believes the disease is peculiar to the lacrymal gland, conjunctiva, and eyelids. Soft cancer, however, or as it is sometives termed, "medullary fungus,"—"fungus hæmatodes," &c. is common to each and every texture, excepting the crystalline and cornea. The early appearances of this formidable disease have been accurately described by Mr. Saunders and Mr. Wardrop. In Mr. Travers' experience it has speedily proved fatal, when arrived at that stage in which the visible enlargement of the ball, livid blue tint of the sclerotica, and distension of the conjunctival and palpebral vessels, present themselves. By these the character of the disease is decided. The staphylomatous protrusions of the sclerotic and choroid coats may be confounded with this disease, unless a careful discrimination be exercised. The complexion, as the disease proceeds, acquires the leaden paleness of cancer, and the rest is broken by deep and lancinating pain. If a child, it is heavy-headed and lethargic, as one affected by hydrocephalus—disturbed by occasional convulsive starts; the stomach often rejecting food; the frame quickly emaciating; and the highest degree of fretfulness and irritability being present. The child usually expires in convulsions. The adult suffers from spasmodic shoots of pain through the head and eyeball, with simultaneous startings on going to rest; but the constitutional disturbance is inconsiderable previous to the protrusion of the fungus, and hæmorrhage, which usually comes on at this period, is exceeding distressing. For numerous pathological details we refer to pages 219-20-21-22.

*Pathology of the Appendages.* Abscess sometimes forms within the orbit, occasioning, anterior to its discharge, an equal protrusion of the globe, with eversion of the palpebræ, dilated pupil, and suspended vision, which is sometimes lost altogether. Adipose and steatomatous tumours also form in the fatty and cellular textures round the eye, turning and fixing it in an opposite direction, and rendering it dim by compression. Mr. Travers has removed them in several instances when projecting over the top, or on one side of the globe. When the conjunctiva is freely divided, the fatty tumour is easily hooked forward, and dissected out with a narrow bistoury; but it is not so easy to remove cysts *entire*, which contain a fluid readily escaping on the least puncture. If part of the cyst be left behind, the wound breaks out again and again.

"The encysted tumour, although it extend to the bottom of the orbit, seldom occasions the distortion of the globe. A disagreeable sense of numbness and coldness affects the integument of the glabella and forehead, after the division of the frontal and supratrochlear nerves; these therefore should be avoided in the operation. The tumor sometimes projects exterior to the tarsus, so as to rise upon the palpebra, but more commonly it is beneath the tarsus and contiguous to the globe. In the former case the cyst lies upon the periosteum of the orbit, and is adherent to it; in the latter it is adherent to the globe." 226.

A case occurred in the London Eye Infirmary, where a cyst, containing hydatids deeply seated in the cavity, protruded the eyeball. "The hydatids were evacuated by a puncture in the cyst; the eye returned into its natural situation, and the patient was completely cured." Our author has related a case of aneurism by anastomosis in the orbit, in the 2d volume of the Medico-Chirurgical Transactions; and Mr. Dalrymple a similar case in the 6th volume of the same work.

Exostoses of the orbit are not common. Our author has never seen them in the living subject of a size to create deformity or material inconvenience. Not so polypi, which in their progress burst through the ethmoid and lacrymal bones, sometimes extruding the eyes, so as to occasion the most horrible deformity. For diseases of the lacrymal gland, see pages 228-9.

*Facial Appendages.* On those little abscesses, denominated "stye," and on warts and vesicles, Mr. Travers makes remarks, and then introduces the subjects of lippitudo, diseased cilia, tinea ciliaris, and trichiasis, for which we must refer to the volume itself. With the following extract respecting ectropion, we shall conclude our analysis of the pathological division of Mr. Travers' work, of which we hope we have presented a fair outline. In our next number we shall analyse the therapeutical and operative portions of a volume which does honour, not only to the author, but to the country to which he belongs.

"The ectropion is the result of injury to the eyelid, as wound, burn, herpetic ulcer, or the sequela of chronic lippitudo. The tarsus of the lower palpebra sometimes falls outward from an apparent loss of elasticity, or the unequal action of the orbicularis muscle. The lid receding from the globe suffers the tears to collect in a pool between them. An unhealthy state of the conjunctiva is, if not the cause, as when villous and redundant, a certain consequence of its eversion and exposure. The case is much aggravated when coadhesion, after burns or neglected wounds, ulceration from any cause, or long enduring eversion, takes place between the skin of the eyelid and cheek. This case admits of palliation, but not of cure. I have much improved several cases by first detaching the fastened lid and forcing it to heal by granulation, and afterwards, removing a triangular portion of the cartilage, according to the proposal of a modern author, for the correction of the eversion, which is the best remedy for such eversions as do not depend upon the protruded conjunctiva.* In this, which is the simplest case of ectropeon, the excision of the diseased conjunctiva is sufficient. Where the everted lid is adherent to the bone, there is a deficiency of cellular substance to produce granulations, and the case is, generally speaking, slightly if at all benefited by operation." 235.

---

### IX.

*General Elements of Pathology.* By WHITLOCK NICHOLL, M.D. M.R.I. A.F.I. of the Royal College of Physicians, London; Member of the Medical and Chirurgical Society; Corresponding Member of the Association of King's and Queen's College of Physicians, Dublin. Octavo, pp. 233. Callow, London, 1820.

DR. PARRY has justly observed in the preface to his Elements of Pathology and Therapeutics. "As the great and

---

* "This remedy results from considering the relative condition of the tarsus and skin at the base of the eyelid, in the two diseases, entropeon and ectropeon. In the first the integument is elongated, in the second

